# Changes in Retinal Pigment Epithelium Related to Cigarette Smoke: Possible Relevance to Smoking as a Risk Factor for Age-Related Macular Degeneration

**DOI:** 10.1371/journal.pone.0005304

**Published:** 2009-04-24

**Authors:** Ai Ling Wang, Thomas J. Lukas, Ming Yuan, Nga Du, James T. Handa, Arthur H. Neufeld

**Affiliations:** 1 Forsythe Laboratory for the Investigation of the Aging Retina, Department of Ophthalmology, Northwestern University School of Medicine, Chicago, Illinois, United States of America; 2 Wilmer Eye Institute, Johns Hopkins University School of Medicine, Baltimore, Maryland, United States of America; University of Oldenburg, Germany

## Abstract

Age-related Macular Degeneration (AMD) is a major cause of central vision loss in the elderly and smoking is a primary risk factor associated with the prevalence and incidence of AMD. To better understand the cellular and molecular bases for the association between smoking and AMD, we determined the effects of Benzo(a)Pyrene (B(a)P), a toxic element in cigarette smoke, on cultured retinal pigment epithelia (RPE) and we examined the RPE/choroid from mice exposed to chronic cigarette smoke. We measured: mitochondrial DNA (mtDNA) damage, phagocytic activity, lysosomal enzymes, exosome markers and selected complement pathway components. In the presence of a non-cytotoxic dose of B(a)P, there was extensive mtDNA damage but no nuclear DNA damage. RPE phagocytic activity was not altered but there were increased lysosomal activity, exocytotic activity and complement pathway components. Retinas from mice exposed to cigarette smoke contained markers for mtDNA damage, exosomes and complement pathway components surrounding Bruch's membrane. Markers for these processes are found in drusen from AMD patients. Thus, smoking may cause damage to mtDNA and increased degradative processes in the RPE. These altered cell biological processes in the RPE may contribute to the formation of drusen in individuals who are cigarette smokers and underlie susceptibility to genetic mutations associated with AMD.

## Introduction

Age-related Macular Degeneration (AMD) is a major cause of loss of central vision in the elderly in the United States. Due to the aging population, the number of people with advanced AMD will increase from 1.75 million, currently, to 3 million by 2020 [1]. Smoking is a primary risk factor associated with the prevalence and the incidence of “dry AMD” and geographic atrophy, in which there is degeneration of the retinal pigment epithelium (RPE), and “wet” AMD, in which there is abnormal vascular cell proliferation and destruction of the RPE and the photoreceptors [2], [3]. The link between cigarette smoking and AMD has been affirmed by both epidemiological and genetic studies [2], [3].

Cigarette smoke, which contains chemical toxins, has been epidemiologically linked with AMD [4]. A review of 17 studies found a two- to threefold increased risk for AMD in current smokers compared with those who never smoked [2], [3]. The association between smoking and AMD has been strengthened even further by recent epidemiologic studies, including the Age-Related Eye Disease Study, which found current smokers were at higher risk and incidence of AMD than both past smokers and those who never smoked [5]. People who stopped smoking more than 20 years earlier were not at increased risk of AMD causing visual loss [4], [6].

In the last several years, human genetics has associated several genes and genetic loci with AMD. Genetic studies have identified a susceptibility locus for AMD, which may be located in or near the hypothetical LOC387715 gene [7], [8]. Kanda et al. have confirmed that this locus is the susceptibility locus for AMD and that this gene encodes a mitochondrial protein [9]. Interestingly, this locus may be associated with smoking in that the combination of the LOC387715 polymorphism and smoking confers a higher risk for AMD than either factor alone [10]. Further evidence for genetic susceptibility related to mitochondria has been provided by Canter et al., who have correlated the mtDNA polymorphism A4917G with AMD [11] and Kimura et al, who showed that a polymorphism in superoxide dismutase 2 is associated with AMD in a small subset of patients [12]. Nevertheless, there is little understanding of the underlying cell biology by which cigarette smoking might contribute to AMD.

Although cigarette smoke contains more than 4000 chemicals, one of the most toxic classes of compounds is the polycyclic aromatic hydrocarbons (PAHs) [13], which form DNA adducts. Benzo(a)Pyrene (B(a)P) is a PAH that forms DNA adducts and has been studied in lung and liver tissue [13]. When mammalian cell cultures are exposed to B(a)P, the extent of covalent modification of mtDNA is far greater than that of nDNA [14]. In vitro experiments with various cell types have routinely used concentrations of 10 to 100 µM. In cultures of bovine RPE cells, Patton et al. found cellular DNA damage and altered morphology at 50 and 100 µM B(a)P [15].

We have previously demonstrated that increased mtDNA damage, autophagy and exosomes in the aged RPE may contribute to the formation of drusen [16], [17]. We now hypothesize that these processes are associated with cigarette smoking and AMD. To better understand the cellular and molecular bases for the epidemiologic and genetic associations between cigarette smoking and AMD, we examined the effects of B(a)P on functions of cultured human RPE (ARPE-19). We also determined whether the RPE/choroid of mice exposed to chronic cigarette smoke developed features of mtDNA damage and increased exocytotic activity that we had seen previously with aging [17].

## Materials and Methods

### APRE-19 cell culture

ARPE-19 cells are a spontaneously transformed human RPE cell line [18]. ARPE-19 cells were purchased from American Type Culture Collection (ATCC, Manassas, VA) and maintained in DMEM/F12 with 10% fetal bovine serum (FBS), according to published methods [19].

### Ethics statement

All animal experiments were conducted according to the ARVO Statement for the Use of Animals in Ophthalmic and Vision Research, and the research was approved by the institutional research board at Johns Hopkins Medical Institutions.

### Viability assay

ARPE-19 cells were seeded at a density of 8000 cells/well onto 96-well plates. One day after seeding, the plate were treated with media containing 0, .25, 2.5, 5, 10, 20, 40, 80 µM B(a)P. After the cells were treated for 24 hr, the cell viability was quantified by MTT assay (Promega, Madison, WI), following the manufacturer's instructions. Briefly, the wells were washed with normal culture media and incubated with MTT for an additional 4 hr at 37°C. Absorbance at 570 nm was determined using a Microplate Reader (Model 680; Bio-Rad, CA). All assay points were determined in triplicate and all experiments were repeated three times.

### Western blot

ARPE-19 cells were lysed in buffer (20 mM HEPES, pH 7.0, 10 mM KCl, 2 mM MgCl2, 0.5% Nonidet P-40, 1 mM Na3VO4, 1 mM PMSF, and 0.15 U ml–1 aprotinin) and homogenized. Protein concentrations were determined using the Bradford colorimetric assay. Thirty micrograms of each protein lysate were loaded in each lane in sample buffer (2% SDS, 10% glycerol, 0.001% bromophenol blue, 1% DTT, and 0.05 M Tris-HCl, pH 6.8), separated on 10% SDS–PAGE (Invitrogen), and transferred to a PVDF membrane (Millipore, Temecula, CA). The blots were blocked with 5% nonfat milk in PBS for 1 hr and incubated with rabbit anti-Cathepsin D (1∶4000, GeneTex), rabbit anti-β glucuronidase (1∶500, Protein Tech Group)), followed by peroxidase-conjugated donkey anti-rabbit IgG (1∶15,000) for 1 hr at room temperature. Finally, the blots were developed by enhanced chemiluminescence (ECL) (Pierce) on Hyperfilm (Amersham). The immunoblots were scanned and relative band density was determined using ImageJ (National Institutes of Health, Bethesda, MD). The densities were normalized to β-actin and analyzed by a standard two-tailed t-test using GraphPad Prism.

### Long Extension-Polymerase Chain Reaction (LX-PCR)

LX-PCR [19] was performed on B(a)P treated ARPE-19 cells (described above). Genomic DNA was isolated with DNeasy Blood & Tissue Kit (Qiagen). The quantitation of the purified genomic DNA, as well as of PCR products, was performed fluorometrically using the PicoGreen dsDNA reagent (Invitrogen). LX-PCR was performed with the GeneAmp XL PCR system (Applied Biosystems), which uses rTth DNA Polymerase XL enzyme designed to amplify target DNA sequences up to about 40 kB. The amounts of primers were 20 pmol and the Mg^2+^ concentration was 1.3 mM. The pairs of PCR primers employed in this study are given in Table 1. All the protocols were initiated by a hot start (75°C, 2 min) prior to addition of rTth enzyme. For amplification of the long fragment of mtDNA, the standard thermocycler program included initial denaturation at 94°C for 1 min, 26 cycles for 15149/14841 or 19 cycles for 5999/14841 of 94°C 15 sec, 65°C 12 min, with final extension at 72°C for 10 min. To amplify a short mtDNA fragment (221 bp) the same program as 15149/14841 was used except that extension temperature was 60°C. To amplify a long nDNA fragment, the thermocycler profile included initial denaturation at 94°C for 1 min, 27 cycles for β-globin or 29 cycles for HPRT of 94°C 15 sec, 65°C 12 min, with final extension at 72°C for 10 min. DNA damage was quantified by comparing the relative efficiency of amplification of large fragments of DNA (16.2 and 7.5 KB from mtDNA and 13.5 and 12.2 KB for nDNA) and normalizing this to the amplification of smaller (221 bp and 84 bp) fragments. The template DNA (1∼50 ng) was varied so that PCR products were obtained during the log phase of the PCR amplification.

**Table 1:** 

A. DNA primers used for LX-PCR		
**16.2-kb mitochondria fragment**		
15149	5′-TGA GGC CAA ATA TCA TTC TGA GGG GC-3′	Sense
14841	5′-TTT CAT CAT GCG GAG ATG TTG GAT GG-3′	Antisense
**8.9 Kb mitochondrial fragment**		
5999	5′-TCT AAG CCT CCT TAT TCG AGC CGA -3′	Sense
14841	5′-TTT CAT CAT GCG GAG ATG TTG GAT GG-3′	Antisense
**Short fragment of mtDNA (221 bp)**		
14620	5′-CCC CAC AAA CCC CAT TAC TAA ACC CA-3′	Sense
14841	5′-TTT CAT CAT GCG GAG ATG TTG GAT GG-3′	Antisense
**13.5-kb fragment from the 5′ flaking region near the â-globin gene**		
48510	5′-CGA GTA AGA GAC CAT TGT GGC AG-3′	Sense
62007	5′-GCA CTG GCT TAG GAG TTG GAC T-3′	Antisense
**10.4 Kb fragment encompassing exons 2–5 of the HPRT gene**		
14577	5′- TGG GAT TAC ACG TGT GAA CCA ACC -3′	Sense
*24997*	*5′- GCT CTA CCC TCT CCT CTA CCG TCC -3′*	*Anti-sense*

B. Primers for real-time RT-PCR	
**CYP1A1**	
5′- AGTGGCAGATCAACCATGACCAGA -3′	Sense
5′- CCGCTTGCCCATGCCAAAGATAAT -3′	Anti-sense
**CFH**	
5′- TGTGTATAAGGCGGGTGAGCAAGT -3′	Sense
5′- ACACAGGAGGTGTCTCTGCATGTT -3′	Anti-sense
**C3**	
5′- TGA CCA CCA GGA ACT GAA CCT TGA -3′	Sense
5′- TCA GCT GTG ACT GTG AAA CCC TCA -3′	Anti-sense
**CFB**	
5′- TGG TTT GGG AAC ACA GGA AGG GTA -3′	Sense
5′- TCC CTT TGA AGG GCG AAT GAC TGA -3′	Anti-sense
**CD59**	
5′- TGA TGG CTG ACA GTG TGT CCA GAT -3′	Sense
*5′- ACT GCT CCC TTC AGA GAA AGC ACA -3′*	*Anti-sense*

### Preparation of photoreceptor outer segments (POS)

POS were isolated according to established protocols from bovine eyes obtained fresh from the slaughterhouse [20]. POS were stored suspended in 10 mM sodium phosphate, pH 7.2, 0.1 M sodium chloride, 2.5% sucrose at −80°C. Before use, POS were thawed and labeled by addition of 20% vol of 1 mg/ml FITC (Molecular Probes) in 0.1 M sodium bicarbonate, pH 9.0, for 1 hr at room temperature in the dark. POS were then washed and re-suspended in cell culture media.

### Phagocytic activity assay

Phagocytosis was measured using a previously published method [21]. Our laboratory previously reported that exposure to fluorescein labeled POS (FITC-POS) to ARPE-19 cells caused a linear uptake for up to 6 hours without loss of cell viability [22]. After exposure to 10 µM B(a)P for 24 hrs, the RPE cells were fed with FITC-POS (10 POS/RPE cell) for 3 hrs under culture conditions before rinsing four times with PBS containing 1 mM MgCl_2_ and 0.2 mM CaCl_2_. The total fluorescence was recorded at 485/525 nm using Tecan plates. Each assay was repeated four times. Intensities were calculated with Graph Pad Prism.

### Cathepsin D enzyme activity

Cathepsin D activity was measured in ARPE-19 cell extracts using a kit containing a fluorogenic peptide substrate peptide, MOCAc-Gly-Lys-Pro-Ile-Leu-Phe-Phe-Arg-Leu-Lys(DNP)-D-Arg-NH2 (Sigma CS0800), reaction buffer (pH 4.0), and standards. Reactions were initiated by the addition of substrate and kinetics of substrate hydrolysis was measured using a fluorescent plate reader (Biotek Synergy 2, Ex 340 nm, Em 460 nm) at 37°C for 15 min with data points collected every 120 sec. Data was imported to Graph Pad Prism for analysis, determination of initial rates, and normalization to total protein assayed.

### N-acetyl- β-glucosoamidase (β glucuronidase activity) enzyme activity

Enzyme activity was measured in ARPE-19 cell extracts using the fluorogenic substrate 4-methylumbelliferyl-N-acetyl- β-glucosoamide (Sigma) and previously published methods [23]. Briefly, cell extract protein (8–10 µg) was added to a pH 4.5 reaction buffer (20 mM Na Acetate, 0.1 M NaCl) in duplicate wells in a 96 well format. The reactions were initiated by adding substrate to a final concentration of 100 µM. Plates were sealed and incubated at 37°C for 60–300 min in a plate reader (Synergy II – BioTek). The generated fluorescence was read at 20 min time intervals (Ex 360 nm, Em 465 nm). Standards of 4-methylubelliferone were used to calibrate the fluorescence signal to nmol of hydrolyzed substrate. Kinetic data was input to Graph Pad Prism for analysis and determination of initial rates.

### Real time RT-PCR

Total cellular RNA from mouse RPE/choroid was isolated and purified (PicoPure™; Arcturus, Mountain View, CA). Samples of the total starting RNA were analyzed by capillary electrophoresis (Agilent Technologies, Palo Alto, CA) to assess the degree of purification. Real time RT-PCR (qRT-PCR) was done using the SYBR-Green dye binding method implemented on an Applied Biosystems 7900 genetic analyzer. Validated primers for each gene of interest were designed for each target mRNA (Table 1B). Optimization of primers and determination of the input cDNA levels were done to ensure appropriate cycle time response. Relative expression was calculated from the differences in cycle time of an internal standard (18 s RNA) compared to the target mRNA.

### Exposure to cigarette smoke

At 8 weeks of age, mice were placed into a smoking chamber for 5 hours/day, 5 days/week for 6 months. This chamber contains a smoking machine (Model TE-10, Teague Enterprises, Davis, CA) that burns 5 cigarettes (2R4F reference cigarettes, 2.45 mg nicotine/cigarette; Tobacco Research Institute, University of Ky) at a time. Eight puffs per minute were taken of 2 seconds duration at a flow rate of 1.05 l/min, to provide a standard puff of 35 cm^3^. The machine is adjusted to produce side stream (89%) and mainstream smoke (11%). The chamber atmosphere is monitored to maintain total suspended particulate at 90 mg/m^3^, and carbon monoxide at 350 ppm. At the rate indicated above, the entire volume of the chamber was exposed to the equivalent of 33 cigarettes/hr. Control mice were kept in a filtered air environment.

### Mouse eye tissues

An equal number of male and female C57Bl6 mice were fed standard rodent chow and water ad libitum, and kept in a 12-hour light-dark cycle. Multiple retinal sections from the eyes of five mice (C57Bl6) exposed to cigarette smoke and eyes of five control mice (C57Bl6) not exposed to cigarette smoke were used. Exposure to cigarette smoke began at 2 months of age and continued for 6 months [24]. Mice exposed to cigarette smoke and control mice were 8 months of age at sacrifice.

### Immunohistochemistry

Cryosections (8 mm) from mice exposed to air or cigarette smoke for 6 months were first blocked with BSA, 5%, for 20 min and incubated at 4°C overnight with a primary antibodies, mouse-anti BPDE-DNA (1∶50, Santa Cruz), mouse anti-CD63, CD81 and LAMP2 (1∶50, Abcam), mouse anti-C3a (1∶50, Chemicon) and mouse anti-C5 (1∶50, Lifespan). Primary antibody was omitted in the negative control. After several washes, tissue sections were incubated with the secondary antibody, anti-mouse rhodamine red (1∶1000, Molecular Probes) for 1 hr at room temperature. After washing with PBS, the slides were mounted with Vectorshield containing DAPI (Vector Laboratory) and observed using confocal microscopy.

### Ultrastructural analysis

After mice were sacrificed and eyes were enucleated, one eye was fixed in 2.5% glutaraldehyde and 1% paraformaldehyde in 0.08 M cacodylate buffer in preparation for electron microscopy. The contralateral eye was either fixed in 2% paraformaldehyde for histochemical analysis. The central 2×2 mm tissue temporal to the optic nerve was postfixed with 1% osmium tetroxide and dehydrated and embedded in Poly/Bed 812 resin (Polysciences, Inc., Warrington, PA). Ultrathin sections were stained with uranyl acetate and lead citrate, and examined with a JEM-100 CX electron microscope (JEOL, Tokyo, Japan) in the Northwestern University Core Facility.

### Statistical analyses

Data are presented as mean±SEM with statistical differences between groups analyzed by standard Student two-tailed t-test and one way ANOVA using GraphPad Prism 5 software. A p value of less than 0.05 was considered statistically significant.

## Results

### mtDNA damage following exposure to B(a)P in ARPE-19 cells

**Figure 1:**
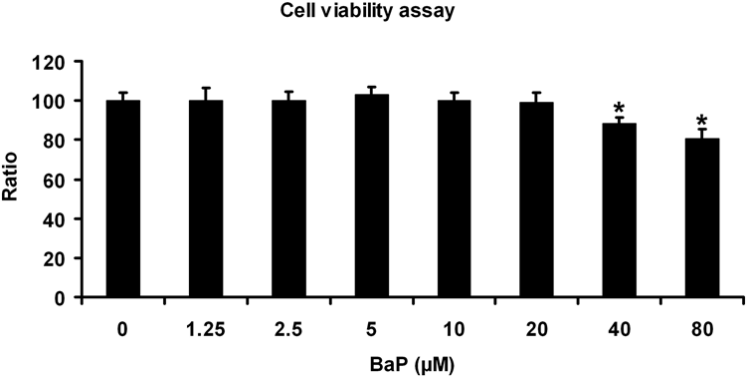
Cell viability assay for ARPE-19 cells treated with B(a)P. Concentrations in the media of 1.25 to 20 µM of B(a)P did not cause a significant decrease of cell viability (p>0.05, n = 10). Data are expressed as normalized ratios (Control = 100).

**Figure 2:**
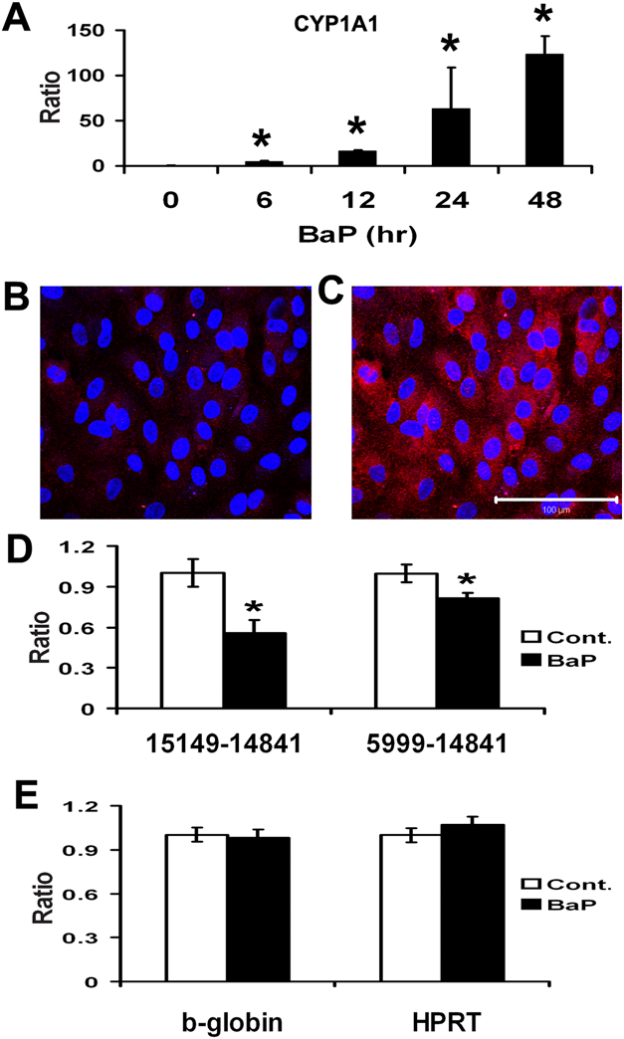
Damage to mtDNA by 10 µM B(a)P. (*A*) real time RT-PCR for CYP1A1. There is a significant increase of CYP1A1 expression levels after 6 hr B(a)P treatment and this increase continued with time at 12, 24 and 48 hrs. (*B–C*) BPDE-DNA adducts in ARPE-19 cells. In these confocal immunofluorescence images, an-anti-BPDE-DNA antibody labels particles in the cytoplasm of ARPE-19 cells after B(a)P treatment (*C*), but not in the control (B). (D–E) LX-PCR for mtDNA and nDNA. B(a)P at 10 µM damaged mtDNA (p<0.05, n = 3)., but not nDNA (p>0.05, n = 3). Data are expressed as normalized ratios (Control = 1).

A dose-response curve was performed to determine a non-cytotoxic dose of B(a)P. We found that B(a)P at a concentration of 10 µM was not cytotoxic to ARPE-19 cells (Fig. 1) and this concentration was used for all further experiments. B(a)P acts as a substrate for the cytochrome P450 1A1 (CYP1A1) isoform and is ultimately converted into B(a)P-7,8-diol 9,10-epoxide (BPDE), which binds covalently to DNA to produce BPDE adducts [13]. We investigated the changes in expression of CYP1A1 when B(a)P was added to the medium using real-time RT-PCR. As shown in Fig. 2A, in the presence of B(a)P, expression of CYP1A1 was significantly increased (p<0.05, n = 3), in RPE cells by 6 hr, and expression increased with time at 12, 24 and 48 hrs. Furthermore, the BPDE-DNA adduct was observed in the cytoplasm of ARPE-19 cells treated with 10 µM B(a)P (Fig. 2C), but not in untreated control cells (Fig. 2B).

Previous studies showed that when cells are exposed to B(a)P, the extent of covalent modification of mtDNA is 40–90 times greater than that of nDNA [14]. We determined whether the effects of B(a)P was primarily on mtDNA or on nDNA in RPE. To assess damage to DNA, we used the Long-extension PCR (LX-PCR) technique [16], [17], [19], [25]. This method determines the relative amplification of long stretches of mtDNA and nDNA (>6 kb), which will be less efficiently transcribed when nucleotides are modified by oxidation or alkylation. We found there was a significant decrease in the relative amplification of mtDNA but not nDNA (Fig. 2D and E), indicating greater B(a)P damage to mtDNA compared to nDNA (p<0.05, n = 3). Agarose gel electrophoresis showed that all mtDNA PCR products were single bands of the appropriate size (data not shown).

### Phagocytic activity

**Figure 3:**
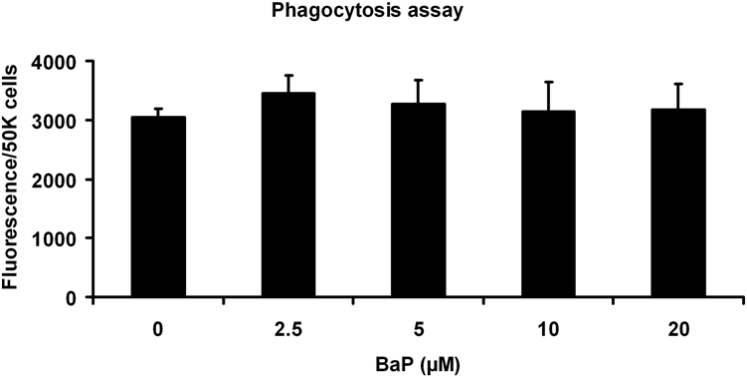
Phagocytic activity of ARPE-19 cells. B(a)P at concentrations in the media of 2.5–20 µM did not change phagocytic activity when cells were exposed to fluorescein labeled bovine photoreceptor outer segments for 3 hr (p>0.05, n = 4).

Functionally, RPE cells are among the most active phagocytic cells in the body [26]. To determine whether B(a)P affects phagocytosis, we measured the phagocytosis of bovine photoreceptor outer segment (POS) that were labeled with fluorescein. ARPE-19 cells were treated with B(a)P for 24 hr and then exposed to fluorescently labeled POS for 3 hr. There was no difference in the fluorescence intensity when cells were treated with B(a)P (Fig. 3). Thus, the damage to mtDNA with 10 µM B(a)P in ARPE-19 cells had no impact on the phagocytosis of POS (p>0.05, n = 4).

### Lysosomal activity

**Figure 4:**
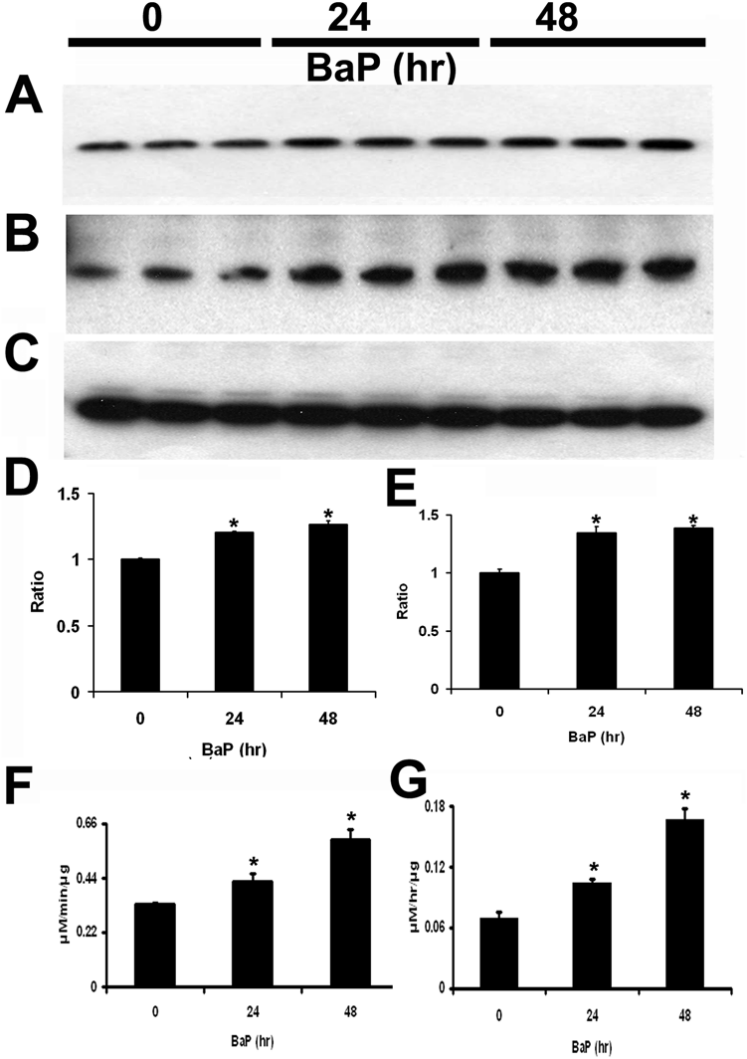
Lysosomal enzyme activity. (A–C) Comparison of protein levels of cathepsin D (A) and β-glucuronidase (B) in ARPE-19 cells by immunoblots. β-actin was used as a loading control (C). (D) Expression of cathepsin D in ARPE-19 cells. The differences in protein levels of cathepsin D were determined by scanning gels and determining the integrated areas of the bands using Image-J software. Data are expressed as normalized ratios to actin. Appropriate background subtraction and normalization of the data to actin was done for each blot. Values are the mean±SEM. There were significant increases in cathepsin D protein following 10 µM B(a)P treatment for 24 hr and 48 hr (p<0.05, n = 3), compared to untreated controls. Data were expressed as normalized ratios to actin. (E) Expression of β-glucuronidase in ARPE-19 cells. The differences in expression levels of β-glucuronidase were determined by scanning gels and determining the integrated areas of the bands using Image-J software. Data are expressed as normalized ratios to actin. Appropriate background subtraction and normalization of the data to actin was done for each blot. Values are the mean±SEM. There were significant increases in β-glucuronidase protein following 10 µM B(a)P treatment for 24 hr and 48 hr (p<0.05, n = 3), compared to untreated controls. (F) Cathepsin D enzymatic activity from cell extracts were significantly increased at 10 µM B(a)P treatment (*p*<0.05, n = 3). (*G*) β-glucuronidase enzymatic activity from cell extracts was significantly increased at 10 µM B(a)P treatment (*p*<0.05, n = 3).

Another major function of the RPE is lysosomal digestion of damaged intracellular macromolecules from autophagy (e.g. arising from damage to mtDNA), phagosomes (containing POS) and endosomes. We determined whether B(a)P affected lysosomal activity. An important lysosomal enzyme in RPE cells is the aspartic protease, cathepsin D [27]. After exposure to 10 µM B(a)P for 24 hr and 48 hr, cathepsin D protein levels (Fig. 4A and D) and activity (Fig. 4F) were significantly increased (p<0.05, n = 3). In addition, we measured another lysosomal enzyme, β-glucuronidase, in RPE. β-glucuronidase protein levels (Fig. 4B and E) and activity (Fig. 4G) were significantly increased after 10 µM B(a)P exposure for 24 hr and 48 hr.

### Exosome markers in stressed RPE

**Figure 5:**
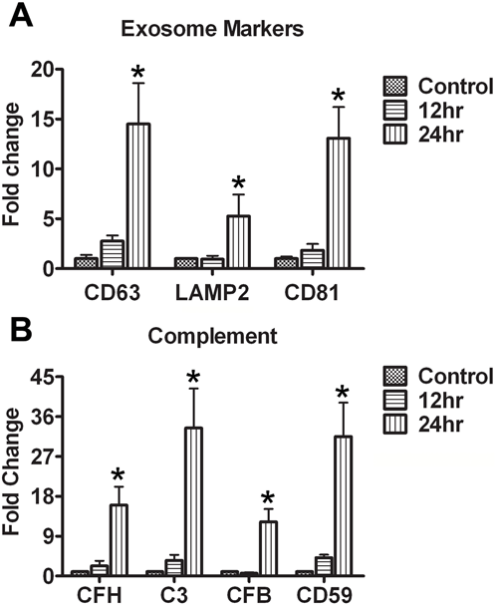
Exosome markers and complement in ARPE-19 cells. (*A*) When the cells were treated with 10 µM B(a)P for 24 hr to damage their mtDNA, gene expression of exosome markers CD63, CD81 and LAMP2 was significantly increased at 24 hrs (*p*<0.05, n = 3). (*B*) When the cells were treated with 10 µM B(a)P for 24 hr to damage their mtDNA, gene expression of complement pathway components CFH, C3, CFB and CD59 was significantly increased at 24 hrs (p<0.05, n = 3).

We hypothesized that RPE, compromised by the toxic components of cigarette smoke, will need to increase removal of damaged intracellular macromolecules. Endosomes, and the exosomes (40–100 nm vesicles) that they form and release, are mechanisms that remove intracellular macromolecules [28]. Thus, we treated ARPE-19 cells with 10 µM B(a)P and measured, by quantitative RT-PCR, three markers for late endosomes and exosomes: CD63, CD81 and LAMP2. As shown in Fig. 5A, exposure to 10 µM B(a)P for 24 hrs caused increased expression of CD63, CD81 and LAMP2 (p<0.05, n = 3).

### Complement activation in stressed RPE

Our previous data, comparing gene expression profiles from young and old mice, showed that the RPE/choroid in the aged mouse has become an immunologically active tissue [29]. We determined the levels of complement pathway components after B(a)P exposure. C3, CFH, CFB are well-known risk factors for AMD. CD59 is upregulated in ARPE-19 cells when treated with oxidized low density lipoprotein [30]. As shown in Fig. 5B, exposure of ARPE-19 cells to 10 µM B(a)P increased expression of C3, CFH, CFB, and CD59 (p<0.05, n = 3). These results are consistent with increased immunological activity in vivo. Thus, in cultured cells, sublethal concentrations of B(a)P induce mtDNA damage, increased lysosomal enzyme activity, increased expression of exosome markers and increased expression of complement pathway components.

### Exosome markers and complement pathway markers in RPE/choroids from mice exposed to chronic cigarette smoke

**Figure 6:**
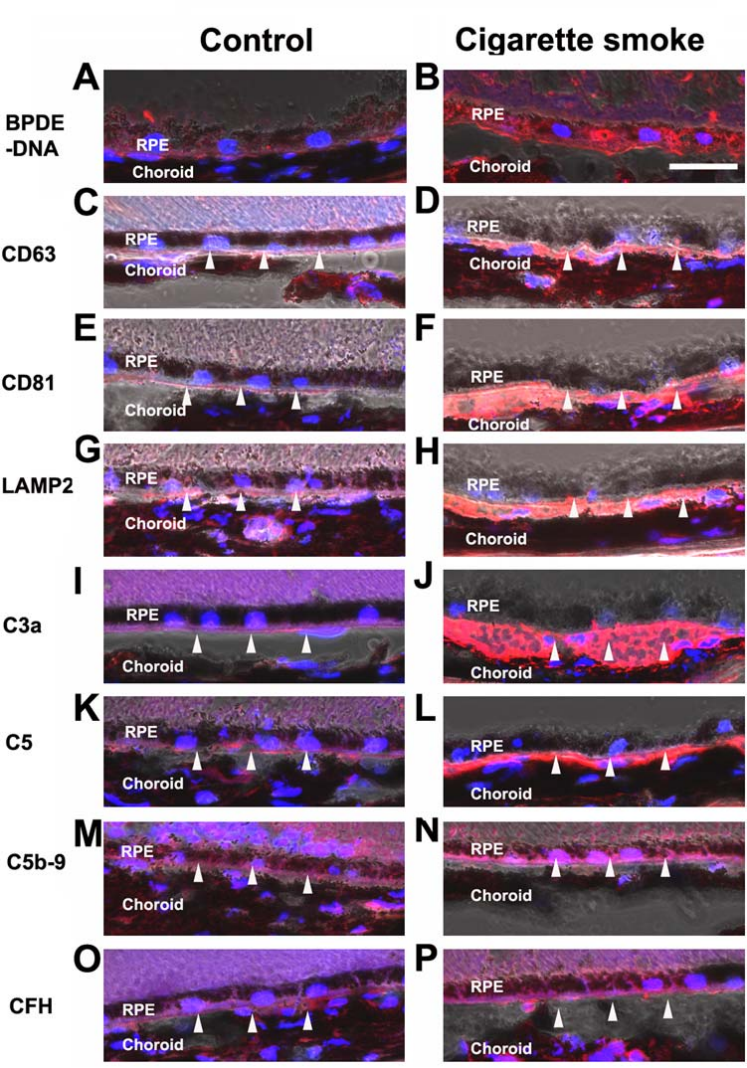
Immunolocalization of BPDE-DNA, exosome markers and complement pathway components in mice exposed to chronic cigarette smoke. These confocal immunofluorescence images were overlayed with the bright field images. (A–B) BPDE-DNA. Immunoreactivity to the BPDE-DNA adduct is present in RPE of mice exposed to chronic cigarette smoke (*B*), but not in control tissues (*A*). (*C–D*) CD63, Immunoreactivity to CD63 is observed in the area of Bruch's membrane of mice exposed to chronic cigarette smoke (*D*), but not in the control tissues (*C*). (*E–F*): CD81. Immunoreactivity to CD81 is observed in the area of Bruch's membrane of mice exposed to chronic cigarette smoke (*F*), but not in the control tissues (*E*). (*G–H*) LAMP2. Immunoreactivity to LAMP2 is observed in the area of Bruch's membrane of mice exposed to chronic cigarette smoke (*H*), but not in the control tissues (*G*). (*I–J*) C3a. Immunoreactivity to C3a is observed in the area of Bruch's membrane of mice exposed to chronic cigarette smoke (*J*), but not in the control tissues (*I*). (*K–L*) C5. Immunoreactivity to C5 is observed in the area of Bruch's membrane of mice exposed to chronic cigarette smoke (*L*), but not in the control tissues (*K*). (*M–N*) Immunoreactivity to C5b-9 is observed in the area of Bruch's membrane of mice exposed to chronic cigarette smoke (*N*), but not in the control tissues (*M*). (*O–P*) Immunoreactivity to CFH is observed in the area of Bruch's membrane of mice exposed to chronic cigarette smoke (*P*), but not in the control tissues (*O*). Scale bar = 20 µm. Blue: DAPI.

To determine whether the effects of B(a)P seen in vitro in ARPE-19 cells are consistent with changes in vivo, we sought evidence for increased exocytotic activity and increased complement pathway components in the RPE/choroid of mice exposed to chronic cigarette smoke for 6 months, starting at 2 months of age. For immunohistochemistry, five mouse eyes were used for each group. Mice exposed to chronic cigarette smoke had positive immunoreactivity to the BPDE-DNA adduct in the RPE, but there was no labeling in control mice (Fig. 6A and B). Interestingly, we found exosome marker proteins, CD63, CD81 and LAMP2, between RPE and choroid from mice exposed to chronic cigarette smoke, but little or no exosome markers were found in the control tissues (Fig. 6C∼H). The exosome markers appeared to be on the choroid-side of Bruch's membrane. We also found that complement pathway components C3a, C5, C5b-9 and CFH were present between RPE and choroid from mice exposed to chronic cigarette smoke, but not in the control tissues (Fig. 6I∼P). These complement pathway components were also on the choroid-side of Bruch's membrane. When sufficient material becomes available, immunoblots should be done to verify these apparent differences.

### Damage to mitochondria in the RPE of mice exposed to cigarette smoke

**Figure 7:**
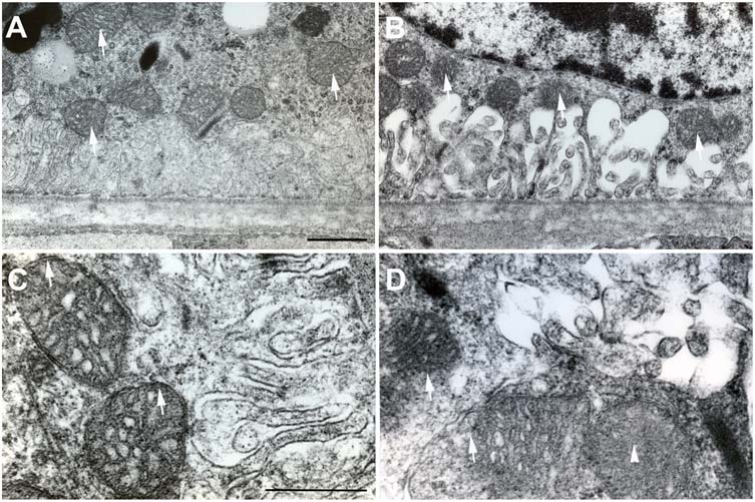
Transmission electron microscopy of the RPE-Bruch's membrane. A. Control, mice exposed to air (n = 5). This representative micrograph shows that mitochondrial membrane and cristae were well preserved and the matrix has homogeneous electron-dense (arrows). B. Mice exposed to cigarette smoke (n = 5). This representative micrograph shows that mitochondria had lost outer membrane and there was disorganization of cristae (arrows). C. Control, mice exposed to air. This representative micrograph at high power shows intact outer membranes of mitochondria. D. Mice exposed to cigarette smoke. This representative micrograph at high power shows loss of mitochondrial outer membranes (arrows) and disorganization of the cristae (arrowhead). A, B: Scale bar = 1 µm; C, D: scale bar = 500 nm.

By electronmicroscopy, the mitochondria in the RPE of 8 months old mice raised in air appeared normal; the membranes and cristae were clearly visible (Fig. 7 A and C). In contrast, the mitochondria in the RPE of 8 months old mice that had been exposed to chronic cigarette smoke exhibited ultrastructural injury. Many mitochondria had lost their outer membranes and had severe disorganization that varied from focal to complete loss of cristae (B and D). The damaged mitochondria were in close proximity to the swollen membrane invaginations on the basal border of the RPE, which has been previously reported (23).

## Discussion

We have previously shown that there is increased mtDNA damage, autophagy and the release of intracellular proteins via exosomes in the RPE of old mice and that many of the markers for these processes are found in drusen in AMD patients [17]. The current results show that B(a)P and cigarette smoke initiates some of the same changes in RPE cells that we found associated with age and AMD. The changes in RPE function that we have found in old mice and now in mice exposed to cigarette smoke, to the extent that such changes occur in humans, are likely to contribute to the cellular and molecular bases for the epidemiologic and genetic associations between cigarette smoking and AMD.

Cigarette smoking is the strongest environmental risk factor associated with AMD. The epidemiologic data, such as the Beaver Dam Eye Study, AREDS study [5], and the Blue Mountain study [31], associate smoking with early AMD as well as progression of AMD. The RPE appears to be a specific target of cigarette smoking associated changes. In human [5], [32] studies, cigarette smoking is associated with RPE abnormalities such as with development of geographic atrophy of the RPE and cell death from apoptosis. Furthermore, mice exposed to chronic cigarette smoke develop evidence of oxidative damage with ultrastructural degeneration of the RPE and Bruch's membrane, as well as RPE apoptosis [24]. Increased oxidative DNA damage to the RPE/choroid has been reported in mice exposed to chronic cigarette smoke [24]. Our findings are consistent with and extend this work by demonstrating that the increased damage was primarily to mtDNA and not nDNA. The ultrastructural changes to RPE mitochondria observed by electronmicroscopy are also consistent with mitochondrial DNA damage.

Increased cathepsin activity has been reported for the human RPE with age [33], [34]. We have previously measured increased expression of cathepsin D in the RPE/choroid of old mice compared to young mice (unpublished data). After treatment with B(a)P, cathepsin D and β-glucuronidase proteins and activities were increased. Thus, B(a)P induced an RPE cell line to increase expression of lysosomal enzymes that are known to increase with age. Whether differential changes in lysosomal enzymes exist in the RPE of AMD patients, comparing smokers and nonsmokers, merits further investigations.

Generation of intracellular damaged macromolecules leads to increased exocytotic activity. Exocytotic activity includes the formation of endosomes, multi-vesicular bodies and the release of exosomes from the cell. Although collection of exosomes from tissue is not possible, our previous studies of mtDNA damage in ARPE-19 cells demonstrated that exosome markers are upregulated and released when the cells are stressed [17]. Treatment of ARPE-19 cells with B(a)P upregulated the same set of exosome markers (CD63, LAMP2, and CD81). Similarly, we found increased exosome markers surrounding Bruch's membrane in the eyes of mice exposed to cigarette smoke, implying that released exosomes and/or their protein components and contents are being trapped locally in the tissue. We previously found that there are exosome markers in drusen from eyes of AMD patients [17]. To the extent that exosomes contribute to the formation of drusen and the subsequent onset of AMD, cigarette smoking may be an accelerating factor for this process. Further studies of AMD patients who were smokers are needed to determine whether cigarette smoking leads to increased exosome markers in the RPE/choroid.

AMD has been associated with local inflammatory responses in the RPE/choroid [35]. In previous work, we demonstrated that the aged RPE/choroid becomes immunologically active [29] due to increased expression of complement components (e.g. C3) and cytokines (e.g. MCP-1) that recruit macrophages and other cells into the tissue. Our model of mtDNA damage in ARPE-19 cells also caused increased release of cytokines [17]. Using B(a)P to cause mtDNA damage in ARPE-19 cells, we now demonstrate that complement components C3, CFH, CFB and CD59 are upregulated. In addition, the RPE/choroid of mice exposed to chronic cigarette smoke had increased expression of complement pathway components such as C3a, C5, C5b-9 and CFH. The increased expression of cytokines and complement pathway components is significant because of genetic polymorphisms associated with increased risk of AMD. For example, a mutation in the HF1/CFH gene increases risk of AMD in both homozygotes and heterozygotes [36]. Similarly, studies of CFB and C2 found variants associated with AMD [37]. Thus, cigarette smoking may further increase the inflammatory activity of the old RPE/choroid and/or promote inflammatory activity in this tissue at an earlier age. Such increased activity in the presence of a gene polymorphism may cause dysfunctional events in the RPE/choroid leading to AMD.

Our findings link B(a)P and cigarette smoke with mtDNA damage, altered lysosomal activity, increased exocytotic activity and complement activation in the RPE. These changes are similar to those seen in aged eyes. Therefore, altered cell biological processes caused by age and/or cigarette smoking may underlie susceptibility to genetic mutations that are found in AMD patients and may be associated with the pathogenesis of AMD in the elderly.
